# Infection by and genotype characteristics of *Enterocytozoon bieneusi* in HIV/AIDS patients from Guangxi Zhuang autonomous region, China

**DOI:** 10.1186/s12879-017-2787-9

**Published:** 2017-10-13

**Authors:** Hua Liu, Zhihua Jiang, Zhongying Yuan, Jianhai Yin, Zunfu Wang, Bingxue Yu, Dongsheng Zhou, Yujuan Shen, Jianping Cao

**Affiliations:** 1 National Institute of Parasitic Diseases, Chinese Center for Disease Control and Prevention; Key Laboratory of Parasite and Vector Biology, Ministry of Health, WHO Collaborating Center for Tropical Diseases, 207 Rui Jin Er Road, Shanghai, 200025 China; 2 Guangxi Zhuang Autonomous Region Center for Disease Control and Prevention, Nanning, 530028 China; 3 Guangxi Medical University, Nanning, 530021 China; 4 Affiliated Hospital of Guilin Medical University, Guilin, 541001 China

**Keywords:** *Enterocytozoon bieneusi*, HIV/AIDS, Genotype, Risk factors

## Abstract

**Background:**

*Enterocytozoon bieneusi* has been increasingly reported to infect humans and various mammals. Microsporidia cause diarrhea in HIV-infected patients worldwide. PCR amplification and sequencing based on the internal transcribed spacer region have been used to describe the genotypes of *E. bieneusi* and transmission of microsporidiosis.

**Methods:**

In this study, we examined *E. bieneusi* infection and genotypes in HIV-positive patients in Guangxi, China. Stool specimens were collected from 285 HIV-positive patients and 303 HIV-negative individuals. *E. bieneusi* genotypes were characterized using nested PCR and sequencing.

**Results:**

Thirty-three (11.58%) HIV-positive patients were infected with microsporidia, and no infection was found in the 303 healthy controls. Three new genotypes were identified and named as GX25, GX456, and GX458; four known genotypes, PigEBITS7, Type IV/K, D, and Ebpc, were also identified. Our data showed that the positive rate for microsporidia was significantly higher in the rural patients than in the other occupation groups. In addition, the positive rate for microsporidia was significantly higher in the patients who drink unboiled water than in those with other drinking water sources.

**Conclusions:**

Our results will provide baseline data for preventing and controlling *E. bieneusi* infection in HIV/AIDS patients. Further studies are required to clarify the epidemiology and potential sources of microsporidia. Our study showed that microsporidium infection occurs in the HIV/AIDS patients in Guangxi, China.

**Electronic supplementary material:**

The online version of this article (10.1186/s12879-017-2787-9) contains supplementary material, which is available to authorized users.

## Background

Gastrointestinal infection is a major opportunistic infection in HIV/AIDS patients, and many studies have reported HIV/AIDS patients co-infected with microsporidia. Microsporidia are obligate intracellular parasites that infect a broad range of vertebrates and invertebrates [1–3]. They have been increasingly recognized as human pathogens in AIDS patients, and they are mainly associated with life-threatening chronic diarrhea and systemic disease [4, 5]. In 1959, the first human case of microsporidiosis was detected, and reports of immunocompromised patients infected by microsporidia have increased [1, 6, 7]. Among the microsporidial species, *Enterocytozoon bieneusi* is the most prevalent human pathogenic species [8]. The infection rate of *E. bieneusi* among HIV patients has been reported to reach up to 50% [9]. Transmission of *E. bieneusi* may involve person-to-person as well as environmental sources, such as ditch water, especially in developing countries with poor sanitation [10, 11]. In addition, zoonotic transmission of *E. bieneusi* has been reported worldwide in various mammal hosts, such as livestock, companion animals, birds, and wildlife. Other routes including waterborn, respiratory or sexual infection have also been reported [12–16].

Considerable genetic variation and genotypes exist within *E. bieneusi* isolates of human and animal origin, and different pathogenic characteristics and host specificity have been found for *E. bieneus*i [3]. Molecular diagnostic methods, especially methods that genotype and subtype pathogens, have been used to characterize the transmission of *E. bieneusi* in HIV patients [17–19]. The internal transcribed spacer (ITS) region of the rRNA gene has been extensively used to identify and describe the genotype characteristics and transmission routes of *E. bieneusi* in humans and animals [20, 21]. To date, more than 204 ITS genotypes have been reported by genotyping analysis, and all the ITS genotypes have been divided into zoonotic (Group 1) and host-specific groups (Groups 2–8) by phylogenetic analysis [22]. Group 1 infects humans and animals, while the other groups are found mostly in specific hosts and wastewater [13, 15, 23]. The presence of the same genotypes of *E. bieneusi* in both humans and animals indicates potential zoonotic transmission [18, 24]. The molecular epidemiologic characterization of *E. bieneusi* has become essential, to predict possible sources of transmission and control the transmission routes.

*E. bieneusi* infection is responsible for 30%–51% of all cases of diarrhea in patients with AIDS [25]. In fact, *E. bieneusi* has been detected in 11.4% and 18.5% of nonhuman primates in Guangxi, and various zoonotic genotypes were identified [14, 26]. Hence, humans, especially HIV patients in Guangxi, could face the risk of *E. bieneusi* infection. To date, no studies have been conducted to describe the *E. bieneusi* infection in HIV or diarrheal patients in Guangxi. In the present study, we aimed to identify the prevalence and genotypes of *E. bieneusi* in HIV-infected patients and case controls in Guangxi and compare the differences between the two groups by using。PCR and sequence analysis of the ITS locus. In addition, we evaluated the public health significance of *E. bieneusi* via phylogenetic analysis and analyzed the risk factors for *E. bieneusi* in the HIV-infected patients on the basis of demographic and clinical data.

## Methods

### Study population

Between July 2013 and July 2014, stool specimens were collected from 285 HIV-positive patients in Guangxi. Among the patients, 216 (75.8%) were males and 69 (24.2%) were females. Most (76.1%) of the participants were farmers and live in rural areas. Demographic data, education level, presence of diarrhea, infective routes, recent CD4^+^ cell counts, and potential risk factors related to waterborne and person-to-person routes and marital status were collected from the participants by attending physicians by using a structured questionnaire at the time of enrollment. The demographic data of the two groups are listed in Table 1. In addition, 303 matched HIV-negative controls with similar demographic and socioeconomic backgrounds were enrolled.

**Table 1:** Risk factors in the occurrence of *Enterocytozoon bieneusi* in HIV/AIDS patients

Risk factor	Number	Infection number	Infection rate (%)	χ^2^	*P* value
Population					
HIV/AIDS	285	33	11.6	37.170	<0.01
Control	303	0	0		
Gender					
Male	216	27	12.5	0.739	0.390
Female	69	6	8.7		
Age group(years)					
< 40	93	12	13.0	0.268	0.875
40–60	113	12	10.7		
> 60	79	9	11.4		
Occupation					
Farmer	217	31	14.3	6.366	0.012*
Others	68	2	2.9		
Education					
Primary	38	2	5.3	3.601	0.165
Middle	123	19	15.4		
Senior	124	12	9.7		
Course of disease					
HIV	32	5	15.6	0.550	0.458
AIDS	253	28	11.1		
CD4^+^ cell count					
CD4 ≥ 200	49	4	8.2	–	1.000
CD4 < 200	119	11	9.2		
HAART treat					
Yes	119	12	9.2	1.48	0.224
No	131	21	13.8		
Transmission route					
Sexual transmission	240	29	12.1	0.274	0.601
Others	45	4	8.9		
Marital status					
Married or cohabiting	214	29	13.6	3.155	0.076
Single	71	4	5.6		
Unboiled water					
Yes	19	5	26.3	4.282	0.039*
No	266	28	10.5		

Note: *Chi-square analysis of different risk factors for the rates of infection *Enterocytozoon bieneusi* by the three parasites; *P* < 0.05

### Specimen collection and DNA extraction

The fecal specimens were preserved in 2.5% potassium dichromate and stored at 4 °C. Aliquots of the stool specimens were shipped to the laboratory. The specimens were collected from patients with fecal excretion heavier than 200 mg and no less than three events of diarrhea per day. Sufficient samples were collected for DNA extraction and purification with the QIAamp DNA Stool Mini Kit (Qiagen, Hilden, Germany). The extracted DNA was stored at −30 °C for PCR and was used for *E. bieneusi* detection and genotyping.

### *E. bieneusi* detection and genotyping

To detect *E. bieneusi*, a 392-bp fragment of the rRNA gene, including ITS, was amplified using nested PCR [27]. Primers used for PCR amplification of ITS gene were listed in Table 2.The amplified fragments were analyzed using agarose gel electrophoresis, and the positive samples were used for sequencing. Genotypes of *E. bieneusi* were determined using sequence analysis of the secondary PCR products and named according to the established nomenclature system. The cycling conditions for *E. bieneusi* were as follows: the primary cycle consisted of 94 °C for 1 min, 35 cycles of 94 °C for 50 s, 56 °C for 30 s and 72 °C for 60 s, followed by 72 °C for 10 min, and termination at 4 °C. A second reaction was carried out similarly. Each specimen was analyzed at least three times by PCR with *E. bieneusi*-positive sample as positive control and nuclease-free water as negative controls in each run, respectively.

**Table 2:** Primers used for PCR amplification of ITS gene

Primer name	Primer sequence(5′-3′)	Fragment size
ITSF1	GATGGTCATAGGGATGAAGAGCTT	
ITSR1	AATACAGGATCACTTGGATCCGT	~410
ITSF2	AGGGATGAAGAGCTTCGGCTCTG	
ITSR2	AATATCCCTAATACAGGATCACT	~390

### DNA sequencing and data analysis

For accurate analysis, all of the genes were amplified at least three times and all PCR-positive products were sequenced in both directions by using the secondary primers with the ABI 3730 DNA Analyzer (Applied Biosystems, Foster City, USA) and Big Dye Terminator v3.1 Cycle Sequencing Kit (Applied Biosystems). ContigExpress was used to evaluate the wave peak and assemble the sequences. All the nucleotide sequences obtained in the present study were searched using the Basic Local Alignment Search Tool, aligned with *E. bieneusi* reference sequences downloaded from GenBank, and analyzed using Clustal X 1.83, MEGA 5 (http://www.megasoftware.net; last accessed in November 2012). Bootstrap analysis with 1000 replicates was used to assess the robustness of the clusters. The chi-square test was used for comparisons.

### Nucleotide sequence accession numbers

The relationship between the *E. bieneusi* genotypes identified in this study and other known genotypes deposited in GenBank was inferred using neighbor-joining analysis of the ITS sequences on the basis of genetic distance by the Kimura two-parameter model. The numbers on the branches are percent bootstrapping values from 1000 replicates. Each sequence was identified by its accession number, host origin, and genotype designation.

Unique nucleotide sequences were deposited in GenBank under the following accession numbers: KP718615 (GX458), KP718616 (GX25), and KP718617 (GX456) [see Additional file 1].

## Results

### Infection rates of *E. bieneusi* in the participants

Of the 285 fecal specimens from the HIV-positive patients, 33 specimens showed positive results for *E. bieneusi* after PCR amplification of the ITS locus. The infection rate of *E. bieneusi* was 11.6% (33/285) in the HIV-positive patients, and *E. bieneusi* was not found in the HIV-negative patients (Table 1). The differences in the infection rates between the HIV-positive and HIV-negative patients were statistically significant different (χ^2^ = 37.17, *P <* 0.01). No age- or sex-associated differences were found in the patients of our study.

### Risk factors for microsporidiosis

In the present study, a number of risk factors related to *E. bieneusi* infection were analyzed, such as gender, age, occupation, water sources, CD4^+^ cell count, marital status, transmission route, and other risk factors (Table 1). The statistical analysis showed that microsporidium infection was significantly associated with the different occupations of the patients. Farmers showed a higher occurrence of microsporidium infection (14.3%, χ^2^ = 6.366, *P* < 0.01) than the other groups with different occupations. In addition, patients who drank unboiled water were more likely to be infected with microsporidia.

### Genotypes of *E. bieneusi*

A total of seven ITS genotypes were obtained from 33 successfully sequenced specimens from the HIV-positive patients. Of them, four genotypes have been previously reported, namely, genotype D (11 cases), type IV/K (seven cases), PigEBITS7 (seven cases), and EbpC (four cases) (Table 3). Three new genotypes were found and named as GX25 (one case), GX456 (one case), and GX458 (one case).

**Table 3:** *Enterocytozoon bieneusi* genotypes in the HIV/AIDS patients in Guangxi, China

Genotype	No. of people infected	Major host
D	11	Humans, Pig, Cattle, Monkey
Type IV/K	8	Humans, Pig, Cat, Monkey
PigEBITS7	7	Humans, Pig, Monkey
Ebpc	4	Humans, Pig, Monkey
GX25	1	Humans
GX456	1	Humans
GX458	1	Humans

### Phylogenetic analysis

**Fig. 1:**
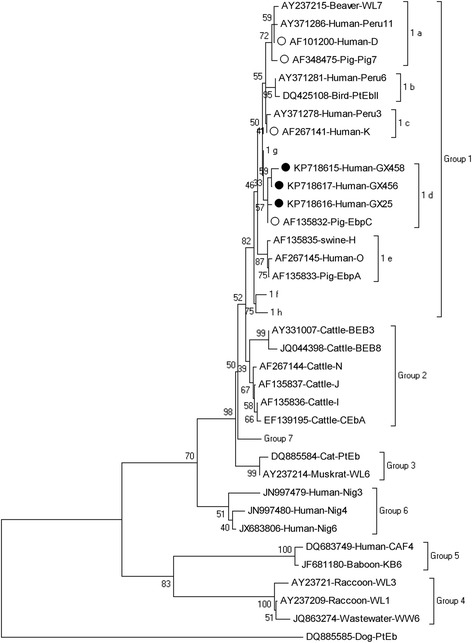
Phylogenetic relationship between the *Enterocytozoon bieneusi* genotype groups. The relationship between the *E. bieneusi* genotypes identified in this study and other known genotypes deposited in GenBank was inferred using neighbor-joining analysis of ITS sequences on the basis of genetic distance by the Kimura two-parameter model. The numbers on the branches are percentage bootstrapping values from 1000 replicates. Each sequence was identified by its accession number, host origin, and genotype designation. The group terminology for the clusters is based on the study by Zhao et al.(2014). The solid and open circles indicate novel and known genotypes identified in this study, respectively

Phylogenetic analysis was performed to understand the genetic relationship among the *E. bieneusi* genotypes. A neighbor-joining tree was constructed using the published *E. bieneusi* ITS nucleotide sequences from humans and domestic animals. These new genotypes were phylogenetically related to Group 1, which contains most of the human pathogenic *E. bieneusi* genotypes (Fig. 1).

## Discussion

PCR and sequence analysis of the ribosomal ITS are regarded as the standard diagnostic technique for identifying and genotyping *E. bieneusi* isolates [24]. To date, the infection rate among HIV-infected patients has been reported to reach up to 50% [28], and *E. bieneusi* causes chronic diarrhea in the patients. In this study, we investigated the prevalence of *E. bieneusi* infection in HIV-infected patients and HIV-negative controls in Guangxi. High prevalence (11.6%, 33/285) of *E. bieneusi* was observed in 285 HIV-positive patients, and *E. bieneusi* was not found in the HIV-negative controls. A previous study conducted on *E. bieneusi* infection in HIV-positive patients in Henan Province showed that the infection rate was 5.7% (39/683) [29]. The difference in the infection rates between the two provinces in China might be attributed to the overall sample size, composition, and health status of the patients, as well as geographical location. In addition, all of the patients in Wang’s study received highly active antiretroviral therapy (HAART), while just fewer than half the patients in this study are receiving HAART. In fact, HAART has been reported to reduce the prevalence of microsporidiosis in HIV/AIDS patients in industrialized nations [30, 31].

In the present study, farmers showed a higher occurrence of microsporidium infection (*P* < 0.01) than the other groups with different occupations. The possible reasons could be the following risk factors: First, the living environment and health conditions in farms are poor when compared with those of the other populations with different occupations. In the countryside, the water used to flush toilets is usually not treated, and many people in these localities do not wash their hands after using the toilet [32]. Therefore, the patients could be infected through fecal-oral transmission. Farmers also have a variety of drinking water sources, such as tap water and pump water, and they provide transmission routes for microsporidia. In fact, a study conducted on the prevalence of intestinal parasitic infections among 463 HIV patients in Benin City, Nigeria, showed that HIV patients who used streams and rivers as sources of water exhibited a significantly higher prevalence of microsporidial infections (*P* = 0.011) [32]. In this study, the patients who drink unboiled water showed a higher microsporidium infection rate (χ^2^ = 4.282, *P* < 0.05) than the other patients. Drinking unboiled water was identified as a risk factor for *E. bieneusi* infection in the present study, which is consistent with the relatively high occurrence of microsporidia in the farmers.

*E. bieneusi* is a major human pathogen associated with chronic diarrhea in HIV-infected patients [33–35]. In a cross-sectional study of zoonotic *E. bieneusi* genotypes in HIV-positive patients on antiretroviral therapy, *E. bieneusi* infection was significantly associated with the occurrence of diarrhea [29]. However, there was no correlation between *E. bieneusi* infection and the clinical symptoms of the HIV-positive patients, which could be mostly attributed to the immune status of the patients and sampling time. In fact, previous studies have found no association between the intensity of microsporidium infection and clinical symptoms [36, 37]. In our study, some other risk factors (age, gender, CD4^+^ level, etc.) and clinical manifestations (diarrhea, white blood cell level, etc.) were also analyzed. However, no correlation was found between these risk factors and *E. bieneusi* infection. Although *E. bieneusi* is nowadays considered to be an opportunistic pathogen in HIV-infected patients or organ transplant recipients, *E. bieneusi* infections have been found in HIV-negative, immunocompetent, and other healthy people [38–41]. In our previous study, *E. bieneusi* was detected using nested PCR in 34 (13.49%) fecal samples from patients with clinical diarrhea in Shanghai [42]. Therefore, detection of *E. bieneus*i is absolutely imperative for HIV-infected patients and individuals with clinical diarrhea.

In this study, three new genotypes and four known *E. bieneusi* genotypes were identified. The new genotypes, namely, GX25 (one case), GX456 (one case), and GX458 (one case), are phylogenetically related to Group 1, which contains most of the human pathogenic *E. bieneusi* genotypes. Sequence alignment and phylogenetic analysis of the *E. bieneusi* isolates on the basis of sequences of the ITS region revealed that the three new genotypes have a high homology with the isolates from pigs (AF135832) [43, 44], indicating their public health significance. The prevalent genotypes were D (11 cases), type IV/K (seven cases), PigEBITS7 (seven cases), and EbpC (four cases). The most frequently observed genotype, D (*n* = 11), has a large variety of hosts and geographic range. It was first detected in humans in Germany then in American, Asian, and African countries [33, 45–54]. In fact, genotype D has been identified in HIV patients [29], animals [15, 26], and wastewater [13] in China. Type IV/K has been detected in HIV patients and non-human primates in Henan Province [29] and cats and dogs in Heilongjiang Province [14]. PigEBITS7, previously found in only pigs [55], has been found in humans [29, 56] and monkeys [15]. EbpC has been detected in HIV-positive and HIV-negative patients [29], pigs [57], and wastewater [13] in China (Table 4). The occurrence of the above-mentioned ITS genotypes in the HIV-positive patients of our study suggest the possibility of zoonotic transmission. This is also supported by the fact that genotype D has been detected in animals in Guangxi [15], and further molecular studies with a large sample size and extensive epidemiological information on humans, animals, and water sources are required to better explain the zoonotic transmission of microsporidiosis.

**Table 4:** Genotypes of *Enterocytozoon bieneusi* in HIV/AIDS patients on the basis of geographical locations worldwide

Geographical area	No. of positive cases/No. of examined cases (%)	Genotype (n)	Reference
Peru	105/2672(3.9)	Peru-1 (35), Peru-2 (18), Peru-3 (1), Peru-4 (1), Peru-5 (3), Peru-6 (1), Peru-7 (8), Peru-8 (4), Peru-9 (9), Peru-10 (3), Peru-11 (6)	[11]
Nigeria (Benin City)	77/463(16.6)	D (31); A (22); TypeIV (14); CAF 2 (2); Eebp A(1); Peru 8 (1); D + IV (1); Nig1 to Nig4 (one each)	[32]
Nigeria (Lagos)	5/90(5.6)	TypeIV (4); one mixed with two unknown genotypes	[10]
Nigeria (Ibadan)	10/132(7.6)	Peru 8 (1); Nig2 (2); new genotype (1); D (1); TypeIV (5);	[48]
Thailand	5/90(5.6%)	D(5)	[33]
Iran	6/15(40)	D (3); E (3);	[49]
Nigeria (Benin City)	18/285(6.3)	Nig4 (2); TypeIV (1); Nig6 (10); Nig7 (2); three with mixed genotypes	[58]
Tunisian^a^	–	D (4);B (2); Peru (1)	[50]
Congo (Kinshasa)	19/242(7.8)	NIA1 (2); D (2); KIN1 (5); KIN2 (5); KIN3 (5);	[51]
Iran	8/356(2.2)	D (−); K (−);	[18]
Cameroon	8/154(5.2)	TypeIV (8);	[59]
Australia (Sydney)	29/159(18.2)	B (29);	[60]
Niamey	24/228(10.5)	A (10); K (1); CAF1 (2); NIA1 (3); D (1);	[53]
Hanoi	3/42(7.1)	D (1); E (1); HAN1 (1)	[53]
Thailand^a^	–	D (12);E (5); PigEBITS7 (4); S (4); Peru (2); O (1); R (1); T (1); U (1); V (1); W (1);	[54]
China (Henan)	39/683 (5.7)	EbpC (18); D (7); TypeIV (6); PigEBITS7 (1); EbpD (1); Peru8 (1); Henan-I to Henan-V (one each)	[29]
Malawi and Netherlands^a^	–	A(1), B(4), C(5), D(6), K(14), S1(2), S2(11), S3(2), S4(1), S5(4), S6(2); S7(1), S8(1), S9(1), 2 unnamed subtypes	[61]
India	–	Lnd1–4	[62]
China (Guangxi)	33/285(11.6)	D (11); TypeIV (8); PigEBITS7 (7); EbpC (1); GX25 (1); GX456 (1); GX458 (1)	The present study

Note: ^a^The sample sizes were not mentioned in the study

## Conclusions

In summary, our study showed the occurrence of microsporidium infection in HIV/AIDS patients in Guangxi, China. The positive rate for microsporidia was significantly higher in the HIV/AIDS patients than in the controls. The four known genotypes indicated that zoonotic transmission of *E. bieneusi* is possible, suggesting that public health education should be provided to prevent and control zoonotic diseases. Three new genotypes of *E. bieneusi* were identified, indicating their public health significance. Our data suggest the possibility of zoonotic transmission of *E. bieneusi* and an association with poor sanitary conditions. Future studies should focus on epidemiological investigations of *E. bieneusi* in various hosts and water sources to better understand the transmission dynamics of microsporidiosis by molecular analysis.

## Additional file

Additional file 1: The data of unique nucleotide sequences. (TXT 1 kb)

## Abbreviations

HAART: Highly active antiretroviral therapy
ITS: Internal transcribed spacer
